# RNA Sequencing Revealed Signals of Evolution From Gallbladder Stone to Gallbladder Carcinoma

**DOI:** 10.3389/fonc.2020.00823

**Published:** 2020-05-29

**Authors:** Jinghan Wang, Chang Xu, Qingbao Cheng, Jiangman Zhao, Shouxin Wu, Wushuang Li, Wencong Ma, Chen Liu, Xiaoqing Jiang

**Affiliations:** ^1^Department of Biliary Tract Surgery I, Third Affiliated Hospital of Second Military Medical University, Shanghai, China; ^2^Shanghai Biotecan Pharmaceuticals Co., Ltd., Shanghai, China; ^3^Shanghai Zhangjiang Institute of Medical Innovation, Shanghai, China

**Keywords:** gallbladder carcinoma, gallbladder stone, RNA sequencing, follow-up time, *ALPP*, *GPR87*

## Abstract

Gallbladder stone is a major risk factor for gallbladder carcinoma (GBC), while there is still a controversy whether period of follow-up since newly diagnoses of asymptomatic gallstones increases the risk of GBC. In this study, 10 GBC patients and 30 patients with gallstones were admitted to our hospital. Patients with gallstones were divided into 3 groups according to the follow-up time, involving 10 patients with follow-up period of 1–3 years (GS3 group), 10 patients with follow-up period of 5–10 years (GS5 group), and 10 patients with follow-up period of more than 10 years (GS10 group). Tumor and para-tumor tissues of GBC patients, and gallbladder tissues of gallstone patients were collected. RNA sequencing was performed on the 50 samples. Besides, 1,704 differentially expressed genes (DEGs) were identified in tumors compared with para-tumor tissues of 10 GBC patients, which were enriched into some well-known cancer-related pathways, such as PI3K-Akt, mitogen-activated protein kinase (MAPK), Ras, and Wnt signaling pathways, and the most significant pathway was neuroactive ligand-receptor interaction. Patients with gallstones with periods of follow-up equal to 1–3 and > 10 years showed to have higher cancer risk than those with 5–10 years. *ALPP* and *GPR87* are potential biomarkers for predicting cancer risk in patients with gallstones. The *in vitro* results revealed that GPR-87 can promote the proliferation, migration, and invasion of GBC cells. Herein, we explored the relationship between GBC patients and patients with gallstones with different periods of follow-up in transcriptome level.

## Introduction

Gallbladder carcinoma (GBC) is a malignancy of hepatobiliary tract, originating from the epithelium mucosa of gallbladder and cystic duct (1), and it ranks sixth among digestive tract malignancy (2). GBC exhibits wide geographical and ethnical variations in its incidence. The incidence and mortality of GBC were 52.8 thousands and 40.7 thousands, respectively, in China, 2015 (3). GBC is a relatively rare disease with a poor clinical prognosis. Its 5-year survival rate was reported to be within 10–20% (4, 5), and its mean overall survival was only 6 months (1). The development of GBC is proposed to occur over a span of 5–15 years, with tissue alterations, including metaplasia, dysplasia, carcinoma *in situ*, and invasive cancer (6). GBC is often diagnosed at advanced stage due to lack of symptoms at early stage (7). The risk factors for GBC include cholelithiasis, chronic cholecystitis (8), genetic susceptibility (9), infection, polyp, biliary tract infections, etc. (10).

As the major risk factor for GBC, gallstone (cholelithiasis) is a very common health problem, globally influencing about 10–20% of adult population, and acute cholecystitis develops in ~20% of patients with biliary colic (11). Gallstones are present in the majority of GBC patients, and previous studies have reported that patients with gallstone have higher risk of GBC than healthy individuals (1, 2, 12). In clinical treatment, laparoscopic cholecystectomy is the first choice for patients with symptomatic gallstones, and annual follow-up is recommended for such patients with asymptomatic gallbladder stones (13).

There is still a controversy whether long-term follow-up of gallstones may increase the risk of GBC. A number of scholars pointed that long-term follow-up of gallstones provides time for chronic inflammation (14), and is associated with an increase in bacterial infection (15), eventually leading to pathological changes. Other researchers found that presence of asymptomatic gallstones didn't increase the risk of GBC (16). Surgery is not merely recommended to prevent GBC for patients with asymptomatic gallstones (17).

In the present study, 10 GBC patients and 30 patients with gallstones with different periods of follow-up were recruited. Tumor tissues and para-tumor tissues of GBC patients and gallbladder tissues of gallstone patients were collected postoperatively. RNA sequencing was performed, and then, bioinformatics analysis was carried out to assess relationship between GBC patients and patients with gallstones with different periods of follow-up in transcriptome level.

## Materials and Methods

### Study Subjects

**Figure 1:**
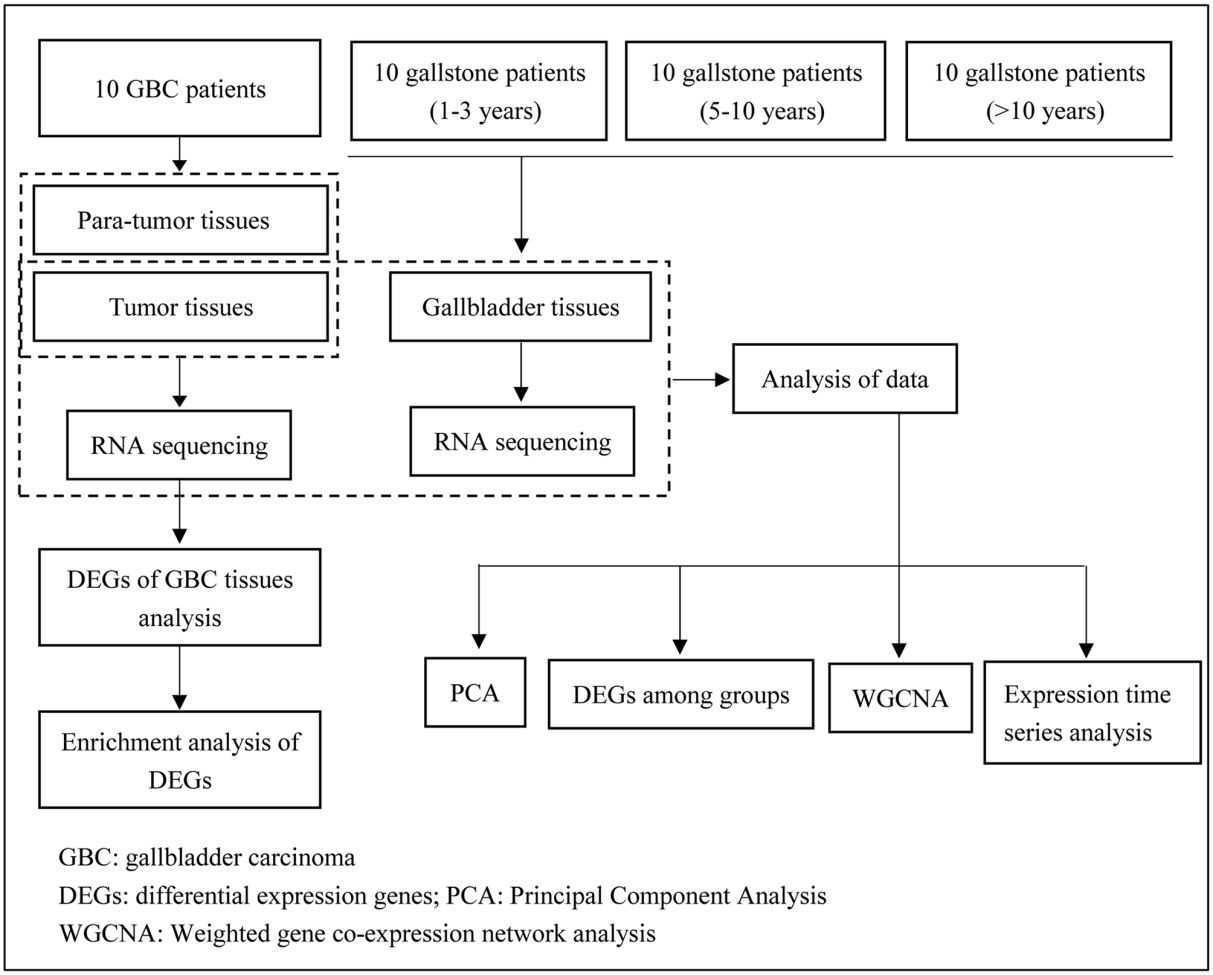
Flowchart of the study process.

In the present study, 10 GBC patients and 30 patients with gallstones were admitted to Department of Biliary Tract Surgery I, The Third Affiliated Hospital of Second Military Medical University (Shanghai, China). Patients with gallstones were divided into 3 groups according to the follow-up time, involving 10 patients with follow-up period of 1–3 years (GS3 group), 10 patients with follow-up period of 5–10 years (GS5 group), and 10 patients with follow-up period of more than 10 years (GS10 group). Clinical characteristics of 10 GBC patients and 30 patients with gallstones are shown in Tables 1, 2, respectively. Fresh tumor tissues and matched para-tumor tissues of 10 GBC patients, as well as gallbladder tissues of 30 patients with gallstones were collected and subjected to RNA sequencing postoperatively (Figure 1). Samples were frozen immediately in liquid nitrogen and stored at −80°C until analysis.

**Table 1:** Clinical characteristics of gallbladder carcinoma patients.

**Clinical characters**	**Patients/*N***
Total	10
Gender	
Male	6
Female	4
Age/years	64.5 (55–76)
Stage	
III	2
IV	8
Tumor size	
>5 cm	4
≤ 5 cm	6
Lymph node metastasis	
Yes	7
No	3
Gallstone	
Yes	7
No	3
Histology	
Adenocarcinoma	7
Adenosquamous carcinoma	3
Differentiation	
Poorly/Poorly and moderately	3
Moderately	7
Smoking	
Yes	2
No	8
Drinking	
Yes	2
No	8

**Table 2:** Clinical characteristics of gallstone patients.

**Clinical characters**	**Patients/*N***
Total	30
Gender	
Male	14
Female	16
Age	58.5 (29–72)
Period of follow-up of gallstone	
1-3 years	10
5-10 years	10
>10 year	10
Mulifocality of stones	
Yes	25
No	5
Size of stone	11.5 (3–31)
Smoking	
Yes	7
No	23
Drinking	
Yes	6
No	24

This study was approved by the Ethics Committee of The Third Affiliated Hospital of Second Military Medical University, and it was conducted in accordance with the Declaration of Helsinki. All subjects signed the written informed consent form prior to commencing the study.

### RNA Extraction and Qualification

Total RNA was extracted from all fresh frozen tissues using RNeasy Mini Kit (Qiagen, Hilden, Germany) according to the manufacturer's protocol. Then, 1% agarose gel was used to monitor RNA degradation and contamination. RNA purity and concentration were measured using the NanoPhotometer® spectrophotometer (IMPLEN, Munich, Germany) and Qubit® RNA Assay Kit in Qubit®2.0 Flurometer (Life Technologies, Carlsbad, CA, USA), respectively. RNA integrity was assessed using the RNA Nano 6000 Assay Kit of the Bioanalyzer 2100 system (Agilent Technologies Inc., Santa Clara, CA, USA).

### RNA-Seq Library Preparation

A total amount of 3 μg RNA per sample was used as input material for the RNA sample preparation. Preparation of non-directional RNA libraries for Illumina sequencing was undertaken using NEBNext® UltraTM RNA Library Prep Kit for Illumina® (Illumina, San Diego, CA, USA) following manufacturer's protocol, and index codes were added to attribute sequences to each sample. Briefly, mRNA was purified from total RNA using poly-T oligo-attached magnetic beads. Fragmentation was carried out using divalent cations under elevated temperature in NEBNext First Strand Synthesis Reaction Buffer (5X). The first strand cDNA was synthesized using random hexamer primer and M-MuLV Reverse Transcriptase. The second strand cDNA synthesis was subsequently performed using DNA Polymerase I and RNase H. After adenylation of 3′ ends of DNA fragments, NEBNext Adaptor with hairpin loop structure were ligated to prepare for hybridization. The library fragments were purified with AMPure XP system (Beckman Coulter Inc., Brea, CA, USA). Then, polymerase chain reaction (PCR) was performed with Phusion High-Fidelity DNA polymerase, Universal PCR primers and Index (X) Primer. Eventually, PCR products were purified by AMPure XP system, and library quality was assessed on the Agilent Bioanalyzer 2100 system (Agilent Technologies Inc., Santa Clara, CA, USA).

### Clustering and Sequencing

The clustering of the index-coded samples was performed on a cBot Cluster Generation System using TruSeq PE Cluster Kit v3-cBot-HS (Illumina, San Diego, CA, USA) according to the manufacturer's instructions. After that, the libraries were sequenced on an Illumina Hiseq platform, and 150-bp paired-end reads were generated.

### Bioinformatics

Raw reads of FASTQ format from RNA-seq data were firstly processed through in-house Perl scripts. In this step, clean data were obtained by removing reads containing adapter, ploy-N, as well as low-quality reads. Simultaneously, Q20, Q30, and GC contents of the clean data were calculated. All the downstream analyses were conducted based on the clean data with high-quality.

The clean reads after quality control were mapped to the human reference genome using HISAT2v2.1.0 program. Then, mapped reads were spliced and merged by StringTie v1.3.3b software. FPKM (Fragments Per Kilobase of transcript per Million fragments mapped) of each gene was calculated to quantify its expression level using StringTie software. We used DESeq2 v1.18 package to identify differentially expressed genes (DEGs) in the two groups (with a false discovery rate (FDR) < 0.05 and |log2FoldChange|≥ 1).

The R package in the Weighted Gene Correlation Network Analysis (WGCNA v1.67) was employed to identify groups of highly correlated genes (18). The power parameter was pre-calculated by the pickSoftThreshold function in WGCNA. In this function, an appropriate soft-thresholding power for network construction was provided by calculating the scale-free topology fit index of several powers. Turning adjacency into topological overlap was carried out, which could measure the network connectivity of a gene defined as the sum of its adjacency with all other genes for the network generation. Hierarchical clustering was performed to classify genes with similar expression profile into modules based on the TOM dissimilarity with a minimum size of 50 for the gene dendrogram. The correlations between the module eigengenes (MEs) and the biological traits were analyzed to identify modules associated with each trait of interest.

To further achieve insight into the function of DEGs and genes in key modules, Gene Ontology (GO) and Kyoto Encyclopedia of Genes and Genomes (KEGG) enrichment analyses were performed by ClusterProfiler 3.6.0 package (19). *P* < 0.05 was considered statistically significant.

### Cell Lines and Cell Culture

The human GBC cell line GBC-SD was obtained from the Cell Bank of the Chinese Academy of Sciences (Shanghai, China). The cell line SGC-996 was kindly supplied by Xinhua Hospital Affiliated to Shanghai Jiao Tong University (Shanghai, China).

GBC-SD was maintained in William's E medium (Gibco, New York, NY, USA), and SGC-996 was cultured in Roswell Park Memorial Institute (RPMI)-1640 medium (Hyclone Laboratories Inc., Logan, UT, USA). All the cells were grown in the medium supplemented with 10% fetal bovine serum (FBS; Gibco, New York, NY, USA) and 1% antibiotics in an incubator with 5% CO_2_ at 37°C.

GPR-87 knockdown sensitized cancer cells were purchased from Shanghai GenePharma (Shanghai, China). GBC-SD and SGC-996 cells were infected with the lentivirus or its control virus, and stable infectants were screened using puromycin, as we previously described.

### Colony Formation Assay

For colony formation assay, GBC-SD shGPR-87 or SGC-996 shGPR-87 and their control cells were separately seeded into 6-well plates (600 cells/well) with 2.5 ml medium, and then maintained at 37 °C in presence of 5% CO2. After cultivation for 7 days, the cells were fixed with 4% paraformaldehyde for 4 h, and then stained with 0.1% crystal violet for 15 min. The number of clones (>50 cells/colony) was counted, and all the assays were repeated for three times.

### Cell Proliferation Assay

Cell proliferation assay was conducted using a Cell Counting Kit-8 (CCK-8; Dojindo, Tokyo, Japan) according to the manufacturer's instructions. Briefly, 1,000 GBC cells in 100 μL medium were seeded into each well of 96-well plates. At the indicated time-points (1, 2, 3, and 4 d), 10 μL CCK8 was added to the each well of the plates, and then incubated for 2 h at 37°C. Next, we measured the absorbance of the plates at wavelength of 450 nm using a microplate reader (Bio-Rad Laboratories Inc., Hercules, CA, USA).

### Immunofluorescence Staining

For immunofluorescence staining, GBC-SD shGPR-87 or SGC-996 shGPR-87 and their control cells were seeded into 96-well plates, and cultured for 48 h. The results were visualized using a Zeiss Axiophot Photomicroscope (Carl Zeiss, Oberkochen, Germany), and analyzed by Image-Pro plus 6.0 software.

### *In vitro* Cell Migration and Invasion Assays

The invasion ability was evaluated by Transwell chamber assay (Corning Life Sciences, New York, NY, USA). The chamber was covered with 50 μl Matrigel Basement Membrane Matrix (2 mg/ml; BD Biosciences, Franklin Lakes, NJ, USA). Serum-free medium (200 μl) containing 2 × 10^4^ cells was added to the upper chamber, while the lower chamber was filled with 600 μl medium supplemented with 15% FBS. Following incubation for 20 h, the cells on the upper chamber were removed, and the remaining cells were fixed with methanol for 20 min, prior to being dyed with 0.1% crystal violet (Sigma-Aldrich, St. Louis, MO, USA). The migration assay was conducted following the aforementioned steps, except for the absence of the Matrigel Basement Membrane Matrix layer, and ~3.5 × 10^4^ cells were added to the upper chamber. Each experiment was performed in triplicate and repeated for 3 times.

### Statistical Analysis

Statistical analyses were performed using SPSS 22.0 (IBM, Armonk, NU, USA) and GraphPad Prism 6 (GraphPad Software Inc., San Diego, CA, USA) software. Data were expressed as mean ± standard devia-tion (SD). Comparisons among groups were carried out with Student's *t*-test. *P* < 0.05 was considered statistically significant.

## Results

### Clinical Characteristics of Patients

Clinical characteristics of 10 GBC patients are presented in Table 1. Among 10 patients, 7 were accompanied by gallstones. Tumors of all GBC patients were at advanced stage (III-IV stage). Clinical factors of 30 patients with gallstones are shown in Table 2.

### Quality Control of Sequencing Data

RNA sequencing achieved an average of 7.71 G clean data per sample with 93.23% mean Q30 after quality control. Within 97.17% of all reads were mapped into reference genome using HISAT2. After quality control of sequencing data, there was no significant difference between the two groups of patients. The quality control demonstrated that RNA sequencing produced high-quality data.

### Differential Expressed Genes (DEGs) in Gallbladder Carcinoma

**Figure 2:**
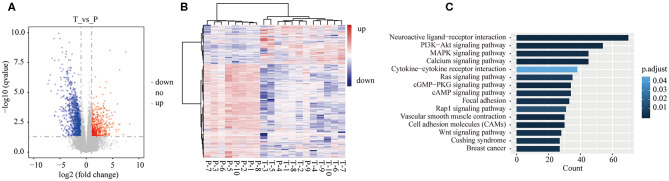
Differentially expressed genes (DEGs) in tumor tissues were compared with para-tumor tissues of 10 GBC patients. **(A)** Volcano plot of the differential expression analysis of genes. **(B)** Heatmap of unsupervised hierarchical clustering of DEGs in all samples of GBC patients. **(C)** The top 15 pathways of DEGs of GBC were identified by KEGG enrichment.

Herein, 1,704 DEGs were identified in tumor tissues compared with para-tumor tissues of 10 GBC patients. Among 1,704 genes, 523 genes were up-regulated, while 1181 genes were down-regulated (Figure 2A, Table S1). The unsupervised hierarchical cluster analysis was performed, which revealed two separate clusters between GBC and para-tumor tissues (Figure 2B). However, it also was unveiled that two para-tumor samples were deflected. P-4 and P-9 samples gathered together with tumor tissues. Besides, GBC patient #4 was in stage IVa, with liver metastasis and lymphatic metastasis of hepatoduodenal ligament, and maximum diameter of tumor was 73 mm. Additionally, GBC patient #9 was in stage IVb, and tumor invaded liver tissue of gallbladder bed. Thus, we speculated that the para-tumor gallbladder tissues of these two patients were invaded by cancer cells.

As shown in Table S1, *ADIPOQ*/adiponectin was the most significant down-regulated gene in tumor tissues (log_2_FoldChange = −13.51, adjusted *P* = 4.16E-23). Adiponectin is expressed in adipose tissue exclusively, and has insulin-sensitizing and anti-inflammatory effects (20). Ogiyama et al.'s study unveiled that lack of adiponectin can promote formation of cholesterol gallstones in mice (21). Chung et al. (22) demonstrated that *ADIPOQ*/adiponectin can inhibit growth of breast cancer cells by inducing cytotoxic autophagy in breast cancer cells through STK11/LKB1-mediated activation of the AMPK-ULK1 axis. The results of the present study indicated that deficiency of *ADIPOQ*/adiponectin may play a potential role in GBC development. *SPRR2E* is the most significant up-regulated gene in tumor tissues (log_2_FoldChange = 30.54, adjusted *P* = 3.40E-09). *SPRR2E* encodes a member of a family of small proline-rich proteins clustered in the epidermal differentiation complex. We found that there were no studies reporting association between *SPRR2E* and cancer, therefore, this gene needs to be further validated in a larger GBC cohort.

### KEGG Enrichment Analysis

To better understand the biological function of the DEGs, KEGG enrichment analysis was performed on 1,704 genes. Among 1704 genes, 674 genes were significantly enriched into 46 pathways (Table S2). Figure 2C shows the top 15 enriched pathways according to adjusted *P*-value. Altered signaling pathways included some well-reported cancer-associated pathways, such as PI3K-Akt (adjusted *P* = 0.0006), mitogen-activated protein kinase (MAPK) (adjusted *P* = 0.002), Ras (adjusted *P* = 0.007), and Wnt (adjusted *P* = 0.003) signaling pathways, and covered focal adhesion (adjusted *P* = 0.003), and cell adhesion molecules (adjusted *P* = 0.0002).

The most significant pathway was neuroactive ligand-receptor interaction (adjusted *P* = 3.69E-10), in which 70 genes were enriched into this signaling pathway. Neuroactive ligand-receptor interaction was frequently found to be altered in gliomas (23–25). In The Cancer Genome Atlas (TCGA), analysis of ovarian carcinoma data showed that neuroactive ligand-receptor interaction ranked fifth among the mutated pathways (26). A previous study pointed out that perineural invasion has a negative influence on survival of GBC patients (27). The results of the current research suggested that neuroactive ligand-receptor interaction pathway may play a significant role in GBC development, although it was not well reported in GBC and other types of cancer.

### Comparing Patients With Gallstones and GBC Patients

**Figure 3:**
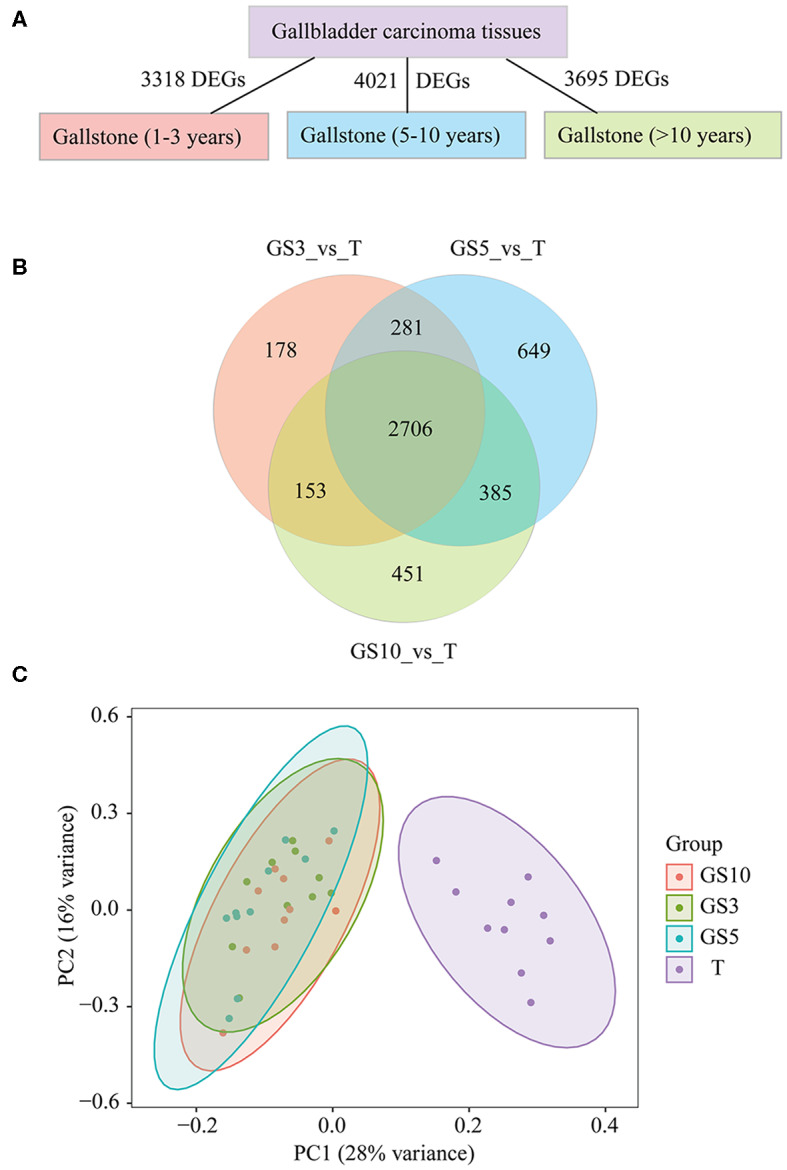
**(A)** Counts of DEGs in GBC tissues were compared with gallbladder tissues of GS3, GS5, and GS10, respectively. **(B)** A Venn diagram of DEGs for making comparison among three groups of T_vs_GS3, T_vs_GS5, and T_vs_GS10. **(C)** Principal Component Analysis (PCA) of four groups of samples, including GBC tissues and gallbladder tissues of GS3, GS5, GS10. GS3: patients with gallstones with period of follow-up equal to 1–3 years; GS5: patients with gallstones with period of follow-up equal to 5–10 years; GS10: patients with gallstones with period of follow-up of >10 years.

To investigate the difference in transcriptome level between patients with gallstones and GBC patients, we analyzed the DEGs in the three groups (GS3, GS5, and GS10) compared with tumor tissues of GBC patients. We found that there were 3318, 4021, and 3695 DEGs in GS3, GS5, and GS10 groups, respectively (Figure 3A). A Venn diagram depicted that 2,706 DEGs were shared among the three groups (Figure 3B), which indicated that patients with gallstones were highly similar in transcriptome level in the three groups. As expected, patients with gallstones gathered together, and those were isolated from GBC patients, as illustrated in PCA diagram (Figure 3C).

**Figure 4:**
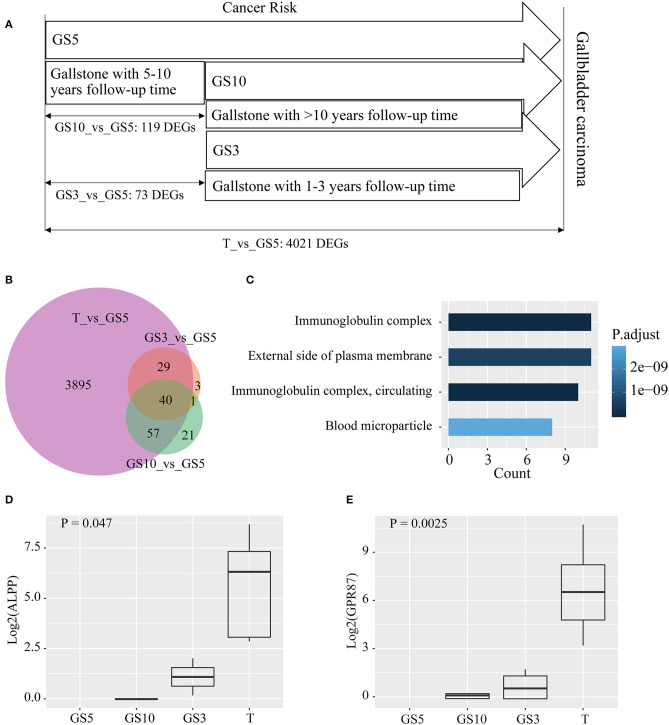
**(A)** Taking GS5 as control, counts of DEGs were compared in tumor tissues of GBC, and gallbladder tissues of GS3 and GS10. **(B)** A Venn diagram of DEGs for making comparison among the three groups. **(C)** Functional analysis of 40 common DEGs in the three groups by GO enrichment. **(D,E)** Expression levels of *ALPP* and *GPR87* in the four groups.

We hypothesized less DEGs between gallstone tissues and tumor tissues indicated more similar with carcinoma tissues in transcriptome level and lower risk of cancer. The results uncovered that cancer risk of gallstones in the three groups from low- to high-level in order was GS5, GS10, and GS3 (Figure 4A), according to counted DEGs. To be pointed, we also observed GS5 group were farther away from GBC than GS3 and GS10, in PCA diagram (Figure 3C), which also provided evidence that GS5 group of gallstone patients had the lowest gallbladder cancer risk.

Taking GS5 patients' tissues as control, 73 and 119 DEGs were identified in GS3 and GS10 groups, respectively (Figure 4A). A Venn diagram (Figure 4B) showed that the majority of DEGs in GS3 and GS10 groups appeared on tumors' DEGs compared with GS5. Besides, almost all the common DEGs exhibited a consistent expression level (up-regulated expression) in GS3 and GS10 groups compared with those in the GS5 group. Table S3 shows the expression levels of 40 common DEGs in the three groups. Results of GO enrichment analysis of the 40 genes are displayed in Figure 4C, including immunoglobulin complex, immunoglobulin complex, circulating, external side of plasma membrane, and blood microparticle (Table S4).

We analyzed expression levels (FPKM) of 40 genes in four groups (GS5, GS10, GS3, and T). We found a number of interesting genes, whose expression levels gradually increased in order from GS5, GS10, and GS3 to T group, which are depicted in Figures 4D,E and Figure S1. Especially, no reads of *ALPP* and *GPR87* were detected in 10 samples of GS5 group (Figures 4D,E). *ALPP* encodes placental alkaline phosphatase, which has been reported as a tumor marker of reproductive system, including ovarian cancer (28, 29), seminoma (30), and testicular germ cell tumors (31). However, an association between *ALPP* and GBC was scarcely studied. Only one case of adenocarcinoma with ectopic human chorionic gonadotropin of the gallbladder was reported, and immunohistochemistry revealed positive staining for placental alkaline phosphatase in tumor cells (32). *GPR87* encodes a G protein-coupled receptor, and Wang et al. reported that overexpression of *GPR87* promotes pancreatic cancer aggressiveness and activates NF-κB signaling pathway (33). *GPR87* promotes cell proliferation in human bladder cancer cells (34), as well as growth and metastasis of CD133+ cancer stem-like cells in hepatocellular carcinoma (35). Other genes that we studied (*IGHV1-24, IGHG1, IGHG4, IGHV3-21, IGHG3, IGHG2*, and *IGHV4-59*) were all enriched into immunoglobulin complex (Figure 4C, Figure S1), which all were immunoglobulin heavy-chain constant-region genes. This result indicated that inflammation plays a pivotal role in carcinogenesis of patients with gallstones.

### WGCNA

**Figure 5:**
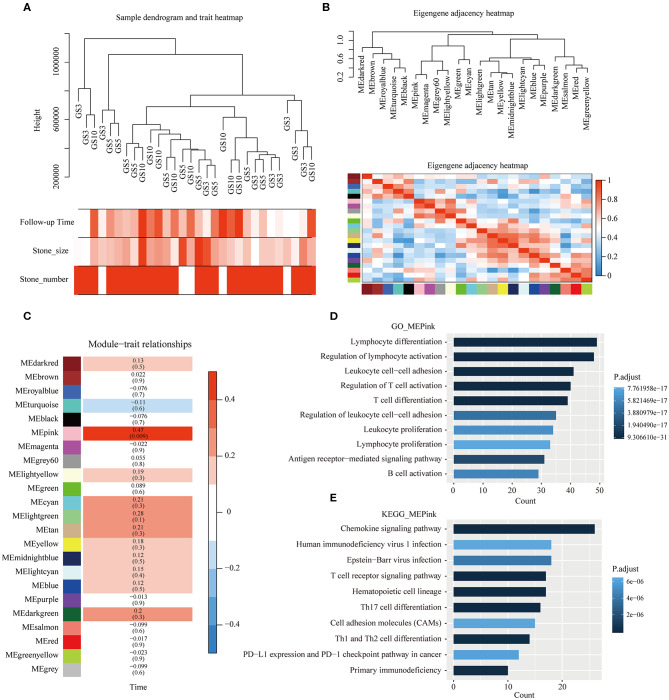
Weighted gene correlation network analysis (WGCNA). **(A)** Clustering of 30 samples and clinical traits of gallstones. The color intensity was proportional to period of follow-up of gallstones, size of stones, and the existence of multiple stones. **(B)** Clustering tree based on modules' eigengenes. **(C)** Associations between modules and period of follow-up of gallstones. Each cell contains the corresponding correlation and P-value, and cells were color-coded by correlation. **(D,E)** Results of GO and KEGG enrichment analyses of genes in pink module.

To further assess the effects of period of follow-up on transcriptome level, we applied WGCNA on 30 patients with gallstones. Figure 5A shows the clustering of 30 samples by fashClust function in WGCNA package, including period of follow-up, size of stone, and the existence of multiple stones. Patients with short period of follow-up were gathered together slightly. Clustering of samples was independent of size of stone and the existence of multiple stones.

Soft-thresholding power is an important parameter for co-expression module, we have performed the analysis of network topology for thresholding powers from 1 to 30. 23 distinct co-expression modules were generated and were shown in different colors (Figure 5B). In general, 23 modules were grouped into four clusters, each module was an independent validation to each other, and presented relative independence of gene expression in each module (Figure 5B).

Correlation between MEs of different modules and period of follow-up was analyzed. The ME of the pink module was highly positively correlated with period of follow-up (correlation coefficient = 0.47, *P* = 0.009) (Figure 5C). Moreover, GO and KEGG enrichment analyses were performed on 306 genes in pink module, which are presented in Figures 5D,E. We found that the majority of functional terms by GO enrichment analysis and a number of signaling pathways by KEGG enrichment were associated with immune cells' behavior, such as lymphocyte differentiation, regulation of lymphocyte activation, T cell receptor signaling pathway, Th17 cell differentiation, etc. The most significant pathway was chemokine signaling pathway. As main drivers of leukocyte recruitment during inflammatory reactions, chemokines act as mediators of alarmins in priming host defense responses after tissue exposure to immune-mediated damage (36).

### *GPR87* Promotes Proliferation of GBC Cells *in vitro*

**Figure 6:**
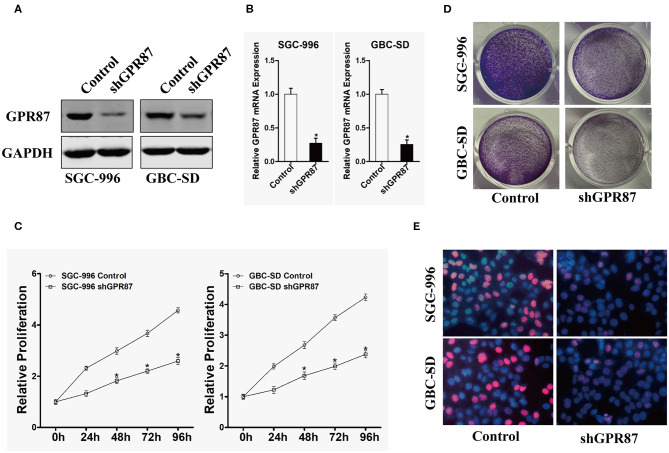
Inhibition of *GPR87* expression attenuates the proliferation of gallbladder cancer cells. **(A)** Western blotting was used to detect the expression level of *GPR87* in gallbladder cancer cells. **(B)** Quantitative PCR was employed to detect the interference efficiency of *GPR87*. **(C)** CCK8 assay was utilized to detect the proliferation ability of gallbladder cancer cells and their control cells after *GPR87* knockdown. **(D)** Clone formation assay was applied to detect the cloning ability of gallbladder cancer cells and their control cells after *GPR87* knockdown. **(E)** Edu experiment detected the proportion of gallbladder cancer cells and their control cells in S-phase after *GPR87* knockdown (*P < 0.05).

To explore the biological role of *GPR87* in the progression of GBC, GBC-SD and SGC-996 cell lines were chosen for stable transfection with shRNA lentivirus vectors toward GPR87. The efficiency of knockdown was verified through Western blotting and quantitative PCR (qPCR), as illustrated in Figures 6A,B.

CCK-8 and colony formation assays were performed to investigate the effects of *GPR87* on the proliferation of GBC cells. As shown in Figure 6C, knockdown of *GPR87* decreased the proliferation of GBC-SD and SGC-996 cells. Consistently, the colony formation capability GBC-SD and SGC-996 cells was significantly suppressed by knockdown of *GPR87* (Figure 6D). EdU test revealed that after knockdown of *GPR87*, the proportion of cancer cells in S-phase was remarkably reduced (Figure 6E). Taken together, the results demonstrated that GPR87 promoted proliferation of GBC cells *in vitro*.

### GPR87 Promotes Migration and Invasion of GBC Cells *in vitro*

**Figure 7:**
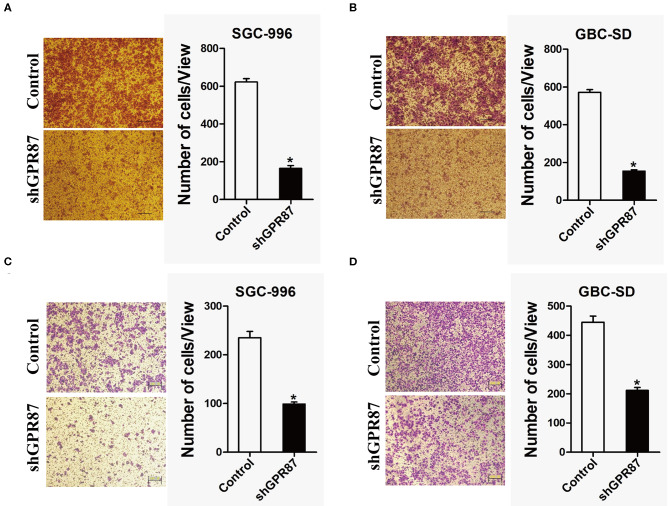
Inhibition of *GPR87* expression attenuates metastatic ability of gallbladder cancer cells. **(A)** Transwell assay was applied to detect the migration ability of shGPR87 SGC-996 and control cells. **(B)** Transwell assay was used to detect the migration ability of shGPR87 GBC-SD and control cells. **(C)** Matrigel invasion assay was applied to detect the invasion ability of shGPR87 SGC-996 and control cells. **(D)** Matrigel invasion assay was employed to detect the invasion ability of shGPR87 GBC-SD and control cells (*P < 0.05).

*In vitro* transwell migration and invasion assays were conducted to investigate the role of *GPR87* in cell migration and invasion. The result showed that cell mobility and invasive capabilities were dramatically attenuated with knockdown of *GPR87* in GBC-SD and SGC-996 cells (Figures 7A–D). Collectively, our data demonstrated that GPR87 promoted migration and invasion of GBC cells *in vitro*.

## Discussion

With the development of sequencing techniques, next-generation sequencing (NGS) was widely applied to various cancer-based studies. However, only a limited number of scholars studied GBC based on omics, such as genome (37) or transcriptome (38) because of its low incidence rate. To better understand the pathogenesis of GBC, we performed RNA sequencing, and identified 1,704 DEGs in tumor tissues compared with para-tumor tissues from 10 GBC patients. Those 1704 DEGs were significantly enriched into 46 signaling pathways, such PI3K-Akt, MAPK, Wnt, and Ras signaling pathways. Neuroactive ligand–receptor interaction was the most significantly altered signaling pathway. Additionally, perineural invasion appeared on the majority of GBC patients, which may be associated with altered neuroactive ligand-receptor interaction signaling pathway. Besides, we observed 45 genes that were enriched into Calcium signaling pathway (adjusted *P* = 1.72E-08). In the present study, among 10 GBC patients, 7 patients accompanied by gallstones. The Ca^2+^-activated small conductance K+ channel appeared to maintain electrical driving force for continued chloride efflux, thereby regulating bicarbonate secretion (39). Hence, it can be concluded that Calciumsignaling pathway is not only associated with gallstone formation, but also influences carcinogenesis.

Chronic inflammation of gallbladder due to presence of gallstone or microbial infection results in sustained production of inflammatory mediators in the tissue microenvironment, which can cause genomic (37) and transcriptome changes (38, 40, 41). Association between gallstones and GBC was almost studied based on epidemiological investigation and pathology, and the molecular mechanism still remains elusive. In the present study, we studied the evolution from different periods of follow-up at transcriptome level using NGS platform. We found that patients with gallstones with middle (5–10 years) period of follow-up had lower cancer risk, while patients with gallstones with short (1–3 years) and long (>10 years) periods of follow-up had more similarity in transcriptome level with GBC patients. For patients with gallstones in GS3 group, because of rapid exacerbation in short-period of time from the first diagnosis, cholecystectomy was performed. Our data indicated that early onset of symptoms for patients with gallstones was an alarm of high-risk of GBC. A middle period of follow-up (<10 years) of gallstones without symptoms can't increase patients' cancer risk. While a long-term follow-up (>10 years) of gallstones may increase patients' cancer risk because of long-term chronic trauma to the mucosa and inflammation stimulation, which was also proved by result of WGCNA. WGCNA identified expression level of a module including 306 genes had significant positive correlation with follow-up time of gallstone, which were enriched into functional terms about immune system such as lymphocyte differentiation, T cell differentiation, leukocyte migration, and so on.

We also found a group of genes' expression levels that were escalated in order from low- to high-risk. We focused on two genes (*ALPP* and *GPR87*). Consistent with the literature reports, the results of the current study suggested that *GPR87* can promote the proliferation and migration of GBC cells. *ALPP* and *GPR87* weren't expressed in tissues in GS5 group, while those were expressed in GS3, GS10 groups. This result indicated that *ALPP* and *GPR87* play a significant role in evolution from gallstones to GBC. *ALPP* and *GPR87* exhibited to have a potential for predicting risk of GBC for patients with gallstones.

Taken together, we profiled transcriptome landscape of GBC using RNA sequencing, and also studied the relationship between GBC patients and patients with gallstones with different periods of follow-up in transcriptome level. We suggest that patients with symptomatic gallstones should accept cholecystectomy as early as possible. It can be concluded that *ALPP* and *GPR87* have a potential to predict cancer risk for patients with gallstones. However, further research should be conducted to identify the relationship between *ALPP* and *GPR87* and their upstream and downstream signaling pathways, and explore their clinical significance as well.

## Data Availability Statement

The datasets generated for this study can be found in the BioProject database (BioProject ID: PRJNA578242, https://www.ncbi.nlm.nih.gov/bioproject/578242).

## Ethics Statement

The studies involving human participants were reviewed and approved by the Ethics Committee of Third Affiliated Hospital of Second Military Medical University. The patients/participants provided their written informed consent to participate in this study.

## Author Contributions

JW, CX and XJ conceived and designed the project. CL enrolled patients. CX and WM collected samples. JZ and SW performed the experiments. WL analyzed the data. JW, CX, and QC contributed to the writing of the manuscript.

## Conflict of Interest

JZ, SW, and WL were employed by the company Shanghai Biotecan Pharmaceuticals Co., Ltd. The remaining authors declare that the research was conducted in the absence of any commercial or financial relationships that could be construed as a potential conflict of interest.

## Abbreviations

GBC: gallbladder carcinoma
DEGs: differentially expressed genes
WGCNA: Weighted Gene Correlation Network Analysis
GO: Gene Ontology
KEGG: Kyoto Encyclopedia of Genes and Genomes
NGS: next-generation sequencing.
