# Supplementary material for: RNA Sequencing Revealed Signals of Evolution From Gallbladder Stone to Gallbladder Carcinoma
Source: Front Oncol. 2020 May 29;10:823. doi: 10.3389/fonc.2020.00823 (PMC7272658; doi:10.3389/fonc.2020.00823)
Supplement: Supplementary file 5 [file Image_1.pdf]

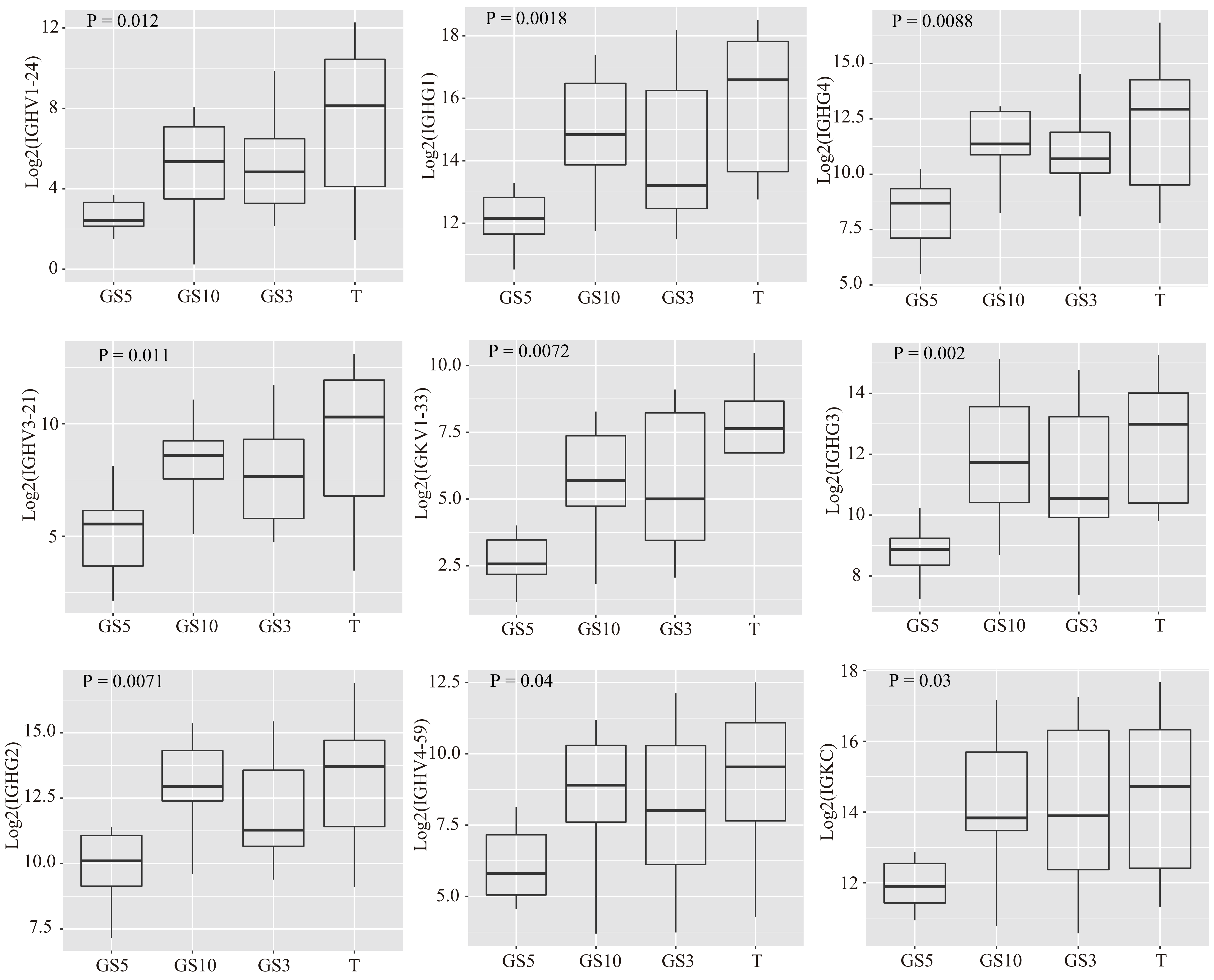

Figure S1 Expression level of 9 genes in four groups which were associated with immunoglobulin complex.
